# Antibiotic susceptibility pattern and resistance genes in *Salmonella* strains isolated from cattle

**DOI:** 10.1186/s12917-025-05081-4

**Published:** 2025-11-14

**Authors:** Muhammad Zeeshan Malik, Saeed Ul Hassan Khan, Hamid Irshad, Ghassan Tayh, Warda Naz, Muhammad Salman Khan, Hanène Belkahia, Mourad Ben Said, Rashid Abbas khan, Mostafa A. Abdel-Maksou, Abdulaziz Alamri, Salman Alrokayan, Aljawharah Fahad Alabbad, Mohamed A. El-Tayeb, Farhad Badshah

**Affiliations:** 1 Department of Zoology, Quaid I Azam University, Islamabad, Pakistan; 2 Animal Health Institute, National Agricultural Research Centre, Islamabad, Pakistan; 3 Laboratory of Microbiology, National School of Veterinary Medicine, University of Manouba, Sidi Thabet, Sidi Thabet 2020, Tunisia; 4 Department of Zoology, Hazara University, Mansehra, KP Pakistan; 5 Department of Zoology, Abdul Wali Khan University Mardan, Mardan, KP Pakistan; 6 Department of Basic Sciences, Higher Institute of Biotechnology, University of Manouba, Sidi Thabet, 2020 Tunisia; 7 Department of Biology, Faculty of Science, Prince of Songkla University, Hat Yai, 90110 Songkhla Thailand; 8 Chair of Biomedical Applications of Nanomaterials, Biochemistry Department, College of Science, King Saud University, P.O. Box 2455, Riyadh, 11451 Saudi Arabia; 9 Biochemistry Department, College of Science, King Saud University, Riyadh, 11451 Saudi Arabia; 10 Botany and Microbiology Department, College of Science, King Saud University, P.O. Box 2455, Riyadh, 11451 Saudi Arabia; 11 Livestock and Poultry Multi-Omics of MARA, Agricultural Genomics Institute at Shenzhen, Chinese Academy of Agricultural Sciences, Shenzhen, 518000 China; 12 State Key Laboratory of Animal Biotech Breeding, Institute of Animal Sciences, Chinese Academy of Agricultural Sciences, Beijing, 100193 China

**Keywords:** Salmonella, Antibiotic resistance, Pakistani cattle, Antibiotic susceptibility, Antibiotic resistance genes, Surveillance

## Abstract

**Background:**

Antibiotic resistance in *Salmonella* strains isolated from Pakistani cattle poses a significant concern for both animal and human health. This study, conducted at the Animal Health Department of the National Agriculture Research Center Islamabad (NARC) and the Zoology Department of Quaid-i-Azam University Islamabad, aimed to determine the antibiotic susceptibility pattern of *Salmonella* isolates recovered from Pakistani cattle and to identify associated antibiotic resistance genes.

**Methods:**

A total of 100 cattle fecal samples suspected of containing *Salmonella* were collected from three commercial dairy farms selected based on owner willingness, prior history of enteric disease outbreaks, and accessibility. The bacterial isolates were processed for *Salmonella* confirmation using growth characteristics, biochemical tests, and *InvA* gene PCR. Antibiotic susceptibility was determined using the disk diffusion method according to CLSI guidelines, and isolates were screened by PCR for antibiotic resistance genes, including *tetA*,* tetB*,* tetC*,* bla*_TEM−1_, *bla*_PSE−1_, *bla*_CMY−2_, *bla*_OXA_,*qnrA*,* qnrB*,* qnrC*,* qnrS*,* tetD*,* tetE*, and *tetG*. Serotyping was not performed.

**Results:**

Out of 100 samples, 28 samples were confirmed as *Salmonella.* Phenotypic testing revealed high resistanceto oxytetracycline (100%) and doxycycline (100%), while ampicillin showed relatively lower resistance (64%). Other antibiotics displayed relatively lower resistance. All isolates were susceptible (100%) to ceftriaxone, cefixime, and sulfamethoxazole-trimethoprim, followed by gentamicin (71%), ciprofloxacin (58%), co-amoxiclav (57%), and imipenem (54%). Among the tested antibiotic resistance genes, *tetA* was highly prevalent which was demonstrated in 25% of the strains, followed by *bla*_TEM−1_ (18%) and *tetC* (3.6%). No positive samples were obtained for *bla*_PSE−1_, *bla*_CMY−2_, *bla*_OXA_, *qnrA*,* qnrB*,* qnrC*,* qnrS*,* tetB*,* tetD*,* tetE*,* or tetG* genes. Several isolates displayed phenotypic resistance without detection of the targeted genes.

**Conclusion:**

This study provides insights into the antibiotic susceptibility pattern and prevalence of antibiotic resistance genes in *Salmonella* isolates from Pakistani cattle. The high resistance to oxytetracycline and doxycycline highlights the need for alternative treatment strategies. However, the findings should be interpreted with caution due to the limited sample size and the absence of serotyping, which restricted identification of specific *Salmonella* serovars. Continued surveillance and targeted control measures remain essential to mitigate the spread of resistant strains.

**Supplementary Information:**

The online version contains supplementary material available at 10.1186/s12917-025-05081-4.

## Introduction

*Salmonella*, a gram-negative, facultative anaerobic bacterium that belonging to the *Enterobacteriaceae* family, is a major pathogen responsible for foodborne illnesses worldwide. It measures approximately 2–3 times 0.4–0.6 microns in size [35, 40]. It is found in the intestine of the livestock, particularly cattle and causing symptoms like fever, abdominal pain, diarrhea, and vomiting in human, typically appearing within 12–72 h after infection [13, 15]. Every year approximately 200 million to 1 billion cases of *Salmonella* infections were reported globally. it has been estimated that approximately 93.8 million cases of gastroenteritis, resulting in 155,000 deaths from acute salmonellosis-related starvation, about 85% of the cases are linked to the consumption of tainted foodChlebicz & Śliżewska [9]),.

In the genus *Salmonella*, there are approximately 2600 identified serotypes, the majority of which have the ability to infect various host animals, including humans and dairy cattle. Infections caused by *Salmonella* can lead to serious food-borne illnesses. In dairy cattle, *Salmonella* infections are commonly associated with early clinical signs of enterocolitis, septicemia, and abortion, with pathogen often reaching the lungs, contributing to severe clinical outcomes [2, 17, 27]. The pathogenicity of *Solmonella* depends on several factors, including the passive transfer of specific immunoglobulins, the infection dose, the age at infection, the immunity developed from previous exposures, and the physical condition of the host [44]. *S. dublin* can act as the primary carrier of the latent stage of the pathogen, which is considered significant for the persistence of bacterial infection in diseased cattle. For instance, pathogenic *S. dublin* has been observed in the gastrointestinal tract of non-shedding cattle [30]. In both adult cattle and calves, the severity of pathogenicity is influenced by factors such as the quantity of inoculum, the level of immunity (whether passive or adaptive), previous exposure to the specific serotype, the virulence of the serotype, and other concurrent stressors affecting the host. It is less likely for the bacterial organism to penetrate the ocular or mucous nasal membranes of cattle [20]. Pathogenic *S. dublin* bacteria can be shed through various routes, including saliva, urine, milk, feces, and vaginal discharge in cattle. There is high variability in the duration and bacterial loads of shedding by diseased cattle. Bacterial pathogens are highly present in animal feces, which serves as the main vehicle for the spread of *S. dublin*. Therefore, fecal samples are commonly analyzed to investigate bacterial shedding in cattle [51].

One concerning characteristic of many *Salmonella* strains is their antibiotic resistance [8]. The development of resistance to at least one antibiotic in three or more different antibiotic categories is defined as multidrug resistance [33]. This type of MDR is particularly concerning as it can be acquired through the consumption of animal-origin foods that were treated with these antibiotics. The misuse and overuse of antibiotics have led to a significant number of MDR *Salmonella* cases worldwide [54]. The pattern of multidrug resistance in *Salmonella* has also extended to broad-spectrum antibiotics such as cephalosporins and fluoroquinolones. The use of antibiotics as growth promoters in agriculture and in veterinary practice to treat animals is considered a major contributor to the spread of antibiotic resistance in *Salmonella*. The risk of zoonotic transmission of MDR *Salmonella* strains through the consumption of contaminated food or water with animal waste is consistently increasing [54]. The use of antibiotics can contribute to the development of resistance, and implementing hygiene measures for the prevention of *salmonellosis* is essential [34]. Despite global attention on antibiotic resistance in *Salmonella*, there is limited information on resistance patterns and the prevalence of specific resistance genes in *Salmonella* isolates from dairy cattle in Pakistan. Understanding these patterns at the farm level is critical for guiding effective treatment strategies and developing interventions to reduce the spread of MDR *Salmonella*. Therefore, the current research aims to investigate the antibiotic susceptibility pattern of *Salmonella* isolates recovered from dairy cattle in Pakistan and to detect associated antibiotic resistance genes in these cattle-associated *Salmonella* isolates.

## Materials and methods

### Ethical considerations

This study was conducted in accordance with ethical guidelines and regulations. Ethical approval was obtained from the Ethical Committee of the Zoology Department, Quaid I Azam University Islamabad, Pakistan (Approval Letter No. APL-ZOL0021913). Informed consent was obtained from the owners of the cattle before collecting the fecal samples.

### Study design

In this study, one hundred fecal samples from cattle suspected of having bovine *salmonellosis*, based on clinical symptoms including diarrhea and fever were collected for analysis. The samples were collected from three dairy farms located in the southern Punjab regions of Pakistan. Farms were selected based on the willingness of farm owners to participate, prior history of enteric disease outbreaks, and accessibility for sampling. Within each farm, cattle were chosen by convenience sampling from among animals showing compatible clinical signs.

### Fecal sample collection

Fecal samples were collected from cattle using sterile gloves to ensure minimal contamination. A total of 10 g of fecal material was collected from each animal. The fecal material was placed in sterile containers immediately after collection to prevent contamination and minimize degradation. The samples were gently mixed to ensure homogeneity before transferring to the laboratory for further analysis.

### Salmonella isolation and identification

The collected fecal samples were transferred to the Bacteriology laboratory for further analysis. The presence of *Salmonella* in the samples was investigated using the following procedure:

each fecal sample (10 g) was pre-enriched by incubating it in buffered peptone water (Oxoid, UK) at 37 °C for 24 h. After the initial incubation, 1 mL of thepre-enriched samples from each sample was transferred and inoculation in selenite cystine broth (10 mL) as a selective enrichment broth of *Salmonella* spp. After incubation, at 37 °C for 24 h, a loop (10 µl) from the enriched samples was streaked onto *Salmonella shigella* agar (SSA) (Oxoid, UK) and incubated overnight at 37 °C on each plate, black colonies were picked up and sub cultured as probably *Salmonella* spp. for further identifications.

### Biochemical characterization using SIM medium

The ability of isolates to produce hydrogen sulfide (H₂S), exhibit motility, and produce indole was assessed using sulfide–indole–motility (SIM) medium. A single colony from a pure culture was inoculated by stabbing the center of the SIM tube with a sterile needle, followed by incubation at 37 °C for 18–24 h. Blackening of the medium indicated H₂S production, diffuse growth from the stab line indicated motility, and the addition of three drops of Kovac’s reagent producing a pink-red color in the upper medium indicated a positive indole reaction.

### Antibiotic susceptibility tests

Antibiotic susceptibility was determined following the method of Bauer et al. (1966) in accordance with Clinical and Laboratory Standards Institute (CLSI) guidelines [53]. Susceptibility to 11 antimicrobial agents was assessed using the disk diffusion (Kirby–Bauer) method on Mueller-Hinton agar (Oxoid, UK). The antibiotic disks and their concentrations were: amoxicillin–clavulanic acid (AUG, 20/10 µg), oxytetracycline (OXY, 30 µg), gentamicin (GEN, 10 µg), imipenem (IMP, 10 µg), ceftriaxone (CRO, 30 µg), cefixime (CFM, 5 µg), ampicillin (AMP, 10 µg), doxycycline (DO, 30 µg), ciprofloxacin (CIP, 5 µg), enrofloxacin (ENR, 5 µg), and trimethoprim-sulfamethoxazole (SXT, 1.25/23.75 µg). Bacterial suspensions were adjusted to the 0.5 McFarland standard, and sterile cotton swabs were used to inoculate the agar surface uniformly. Antibiotic disks were placed aseptically, and plates were incubated at 37 °C for 24 h. Zone diameters were measured and interpreted according to CLSI breakpoints. Isolates resistant to at least one antimicrobial agent in three or more antibiotic classes were classified as multidrug resistant (MDR).

### Genomic DNA isolation and molecular detection of Salmonella

Genomic DNA was extractedfrom the *Salmonella* isolates using a commercial DNA extraction kit (Thermo Fisher) following the manufacturer’s instructions. Briefly, bacterial cells were lysed, and DNA was purified using column-based purification. The quality and quantity of the extracted DNA were assessed using spectrophotometry. Polymerase Chain Reaction (PCR) was performed to detect the presence of *Salmonella*-specific genes in the extracted DNA. *Salmonella* spp. isolates were verified by amplifying the *invA* gene which is genus-specific, and the *iroB* gene for the identification of *S. enterica* using appropriate primers, as previously documented [41]. The PCR reaction mixture contained template DNA, specific primers, dNTPs (deoxynucleotide triphosphates), and DNA polymerase. The PCR amplification was carried out in a thermal cycler (Thermo Fisher) with the following cycling conditions for *invA*: initial denaturation at 95 °C for 5 min, followed by 35 cycles consisting of 95 °C for denaturation for 30 s, annealing at a specific temperature 61°Cfor 30 s, and extension at 72 °C for 1 min. A final extension step at 72 °C for 5 min was included. The PCR condition was same for *iroB* gene using different annealing temperature of 55 °C.The PCR products were separated using agarose gel electrophoresis (1.5%). The amplified DNA fragments were separated based on their size using DNA Ladders, and the presence of the target genes were visualized under UV light after staining the gel with a DNA-specific dye. A positive result indicated the presence of *Salmonella* DNA, confirming the identification of the isolates. Bacterial strains carrying the target genes, previously isolated from animals in our laboratory, were used in this study as positive controls for antibiotic resistance genes or virulence genes.

### Detection of antibiotic resistance genes

To further investigate the antibiotic resistance mechanisms in *Salmonella* isolates, the presence of specific antibiotic resistance genes was determined. The antibiotic resistance genes were studied in the extracted DNA using specific primers targeting (Table 1) by PCR amplification, including genes confer resistance to tetracycline [*tetA*,* tetB*,* tetC*,* tetD*,* tetE*,* tetG*], beta-lactam [*bla*_*TEM−1*_, *bla*_*PSE−1*_, *bla*_*CMY−2*_, *bla*_*OXA*_], and quinolones [*qnrA*,* qnrB*,* qnrC*,* qnrS*]. The amplification products were visualized on an agarose gel electrophoresis. The presence or absence of the target genes was determined based on the size of the amplified fragments.

**Table 1:** List of *Salmonella* primers for antibiotic resistant genes

Gene	Primer name	Sequence (5′ → 3′)	Fragment size (bp)	References
*bla*_*PSE*_	Sal-blaPSE-1-F	CGCTTCCCGTTAACAAGTAC	419	[31]
	Sal-blaPSE-1-R	CTGGTTCATTTCAGATAGCG		
*bla*_*CMY−2*_	Sal-blaCMY-2-F	TGGCCAGAACTGACAGGCAAA	462	[39]
	Sal-blaCMY-2-R	TTTCTCCTGAACGTGGCTGGC		
*bla*_*TEM−1*_	Sal-blaTEM-1-F	AGGAAGAGTATGATTCAACA	535	[52]
	Sal-blaTEM-1-R	CTCGTCGTTTGGTATGGC		
*bla*_*OXA*_	Sal-blaOXA-F	ACCAGATTCAACTTTCAA	589	[28]
	Sal-blaOXA-R	TCTTGGCTTTTATGCTTG		
*qnrA*	Sal-qnrA-F	AGAGGATTTCTCACGCCAGG	580	[7]
	Sal-qnrA-R	TGCCAGGCACAGATCTTGAC		
*qnrB*	Sal-qnrB-F	GATCGTGAAAGCCAGAAAGG	469	[6]
	Sal-qnrB-R	ATGAGCAACGATGCCTGGTA		
*qnrC*	Sal-qnrC-F	GGGTTGTACATTTATTGAATCG	307	[22]
	Sal-qnrC-R	CACCTACCCATTTATTTTCA		
*qnrS*	Sal-qnrS-F	GCAAGTTCATTGAACAGGGT	428	[7]
	Sal-qnrS-R	TCTAAACCGTCGAGTTCGGCG		
*tetA*	Sal-tetA-F	TTGGCATTCTGCATTCACTC	494	[1]
	Sal-tetA-R	GTATAGCTTGCCGGAAGTCG		
*tetB*	Sal-tetB-F	CAGTGCTGTTGTTGTCATTAA	571	[1]
	Sal-tetB-R	GCTTGGAATACTGAGTGTTAA		
*tetC*	Sal-tetC-F	CTTGAGAGCCTTCAACCCAG	418	[1]
	Sal-tetC-R	ATGGTCGTCATCTACCTGCC		
*tetD*	Sal-tetD-F	GCAAACCATTACGGCATTCT	546	[1]
	Sal-tetD-R	GATAAGCTGCGCGGTAAAAA		
*tetE*	Sal-tetE-F	TATTAACGGGCTGGCATTTC	544	[1]
	Sal-tetE-R	AGCTGTCAGGTGGGTCAAAC		
*tetG*	Sal-tetG-F	GCTCGGTGGTATCTCTGCTC	550	[1]
	Sal-tetG-R	CAAAGCCCCTTGCTTGTTAC		

### Data analysis

The collected data on *Salmonella* isolation, identification, antibiotic susceptibility, and presence of antibiotic resistance genes were analyzed using appropriate statistical methods in Statistical Package for the Social Sciences (SPSS, version26). Statistical significance was setat a *p-*value of less than 0.05.

### Limitations

It is important to acknowledge the limitations of this study. The sample size was limited to 100 fecal samples from cattle suspected of Salmonellosis which have diarrhea and fever, which may not represent the entire population. Additionally, the study focused on a specific geographical area and may not be generalizable to other regions. Furthermore, the isolates were not serotyped to determine specific Salmonella serovars. While genus-level confirmation provided valuable insights into the overall prevalence and resistance patterns, serotyping could have offered more detailed epidemiological information, aiding in source tracking and targeted control strategies. The findings should be interpreted within these limitations.

## Results cultural and morphological characterization

**Fig. 1:**
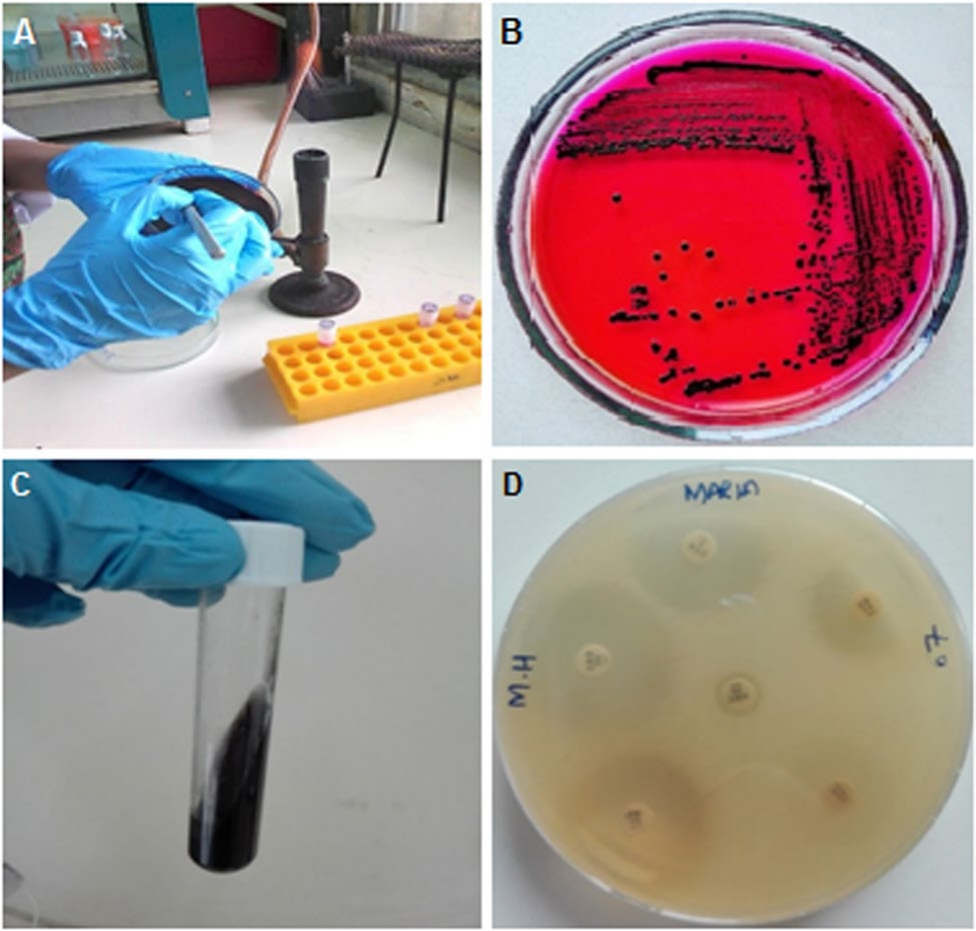
*Salmonella*culture purification & biochemical and antibiotic susceptibility testing. Legend:** A** *Salmonella* primary culturing, **B** *Salmonella* secondary culture (purified), **C** Hydrogen sulfide production test, D. Disk diffusion test for antibiotic susceptibility

Out of the hundred suspected fecal samples, 28 isolates of *Salmonella* were recovered by culturing on *Salmonella Shigella* agar (SSA). The colonies presented as black on SSA, confirming their identification as *Salmonella.* The black coloration of colonies is a characteristic indicator of *Salmonella*, resulting from the production of hydrogen sulfide (H₂S) gas (Fig. 1a and b).

### Biochemical characterization

After bacterial identification based on observation morphology, the 28 suspected *Salmonella* isolates were subjected to biochemical tests for further confirmation. All isolates showed the presence of black color in the SIM tubeswhich acts as an indicator for the presence of hydrogen sulphide gas (Fig. 1c).

### Confirmation by PCR

**Fig. 2:**
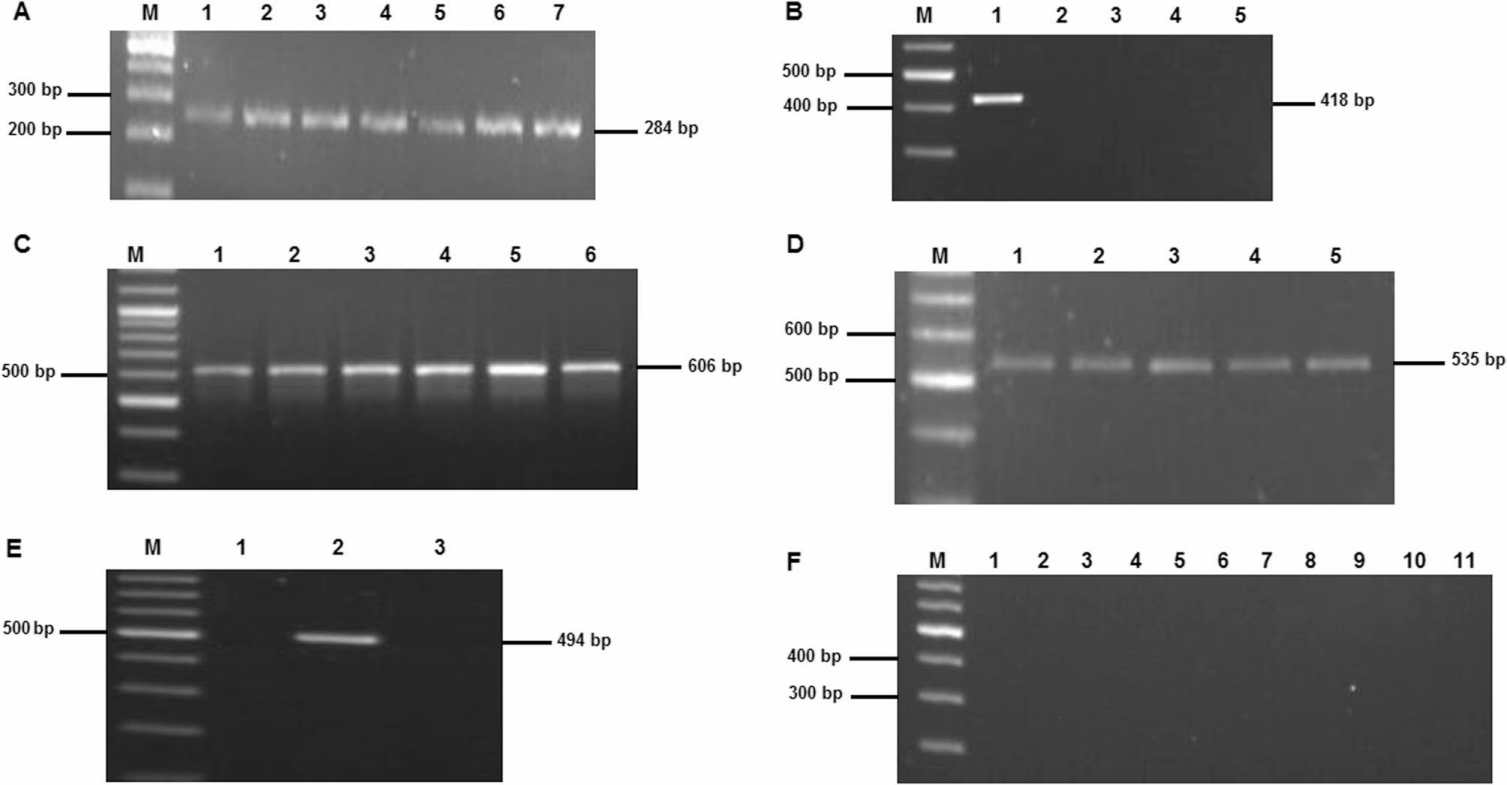
Legend: Lane M = Ladder 100 bp; Agarose gel concentration: 1.5%. **A** InvA gene detection, **B** tetC gene detection, **C** IroB gene detection, **D** blaTEM-1 gene detection, E. tetA gene detection, F. no gene detected for blaPSE-1, blaCMY-2, blaOXA, qnrA, qnrB, qnrC, qnrS, tetB, tetD, tetE, and tetG

All 28 isolates were subjected to PCR targeting the *InvA* gene for molecular confirmation. A band of the expected size (284 bp) was observed in all *Salmonella* isolates, confirming their identification (Fig. 2A). Additionally, PCR targeting the *iroB* gene was performed to assess the presence of this gene in the isolates. A band of the expected size (606 bp) was observed in all 28 isolates (Fig. 2C), confirming the presence of the iroB gene in all *S. enterica* isolates.

### Antibiotic sensitivity profile determination

**Fig. 3:**
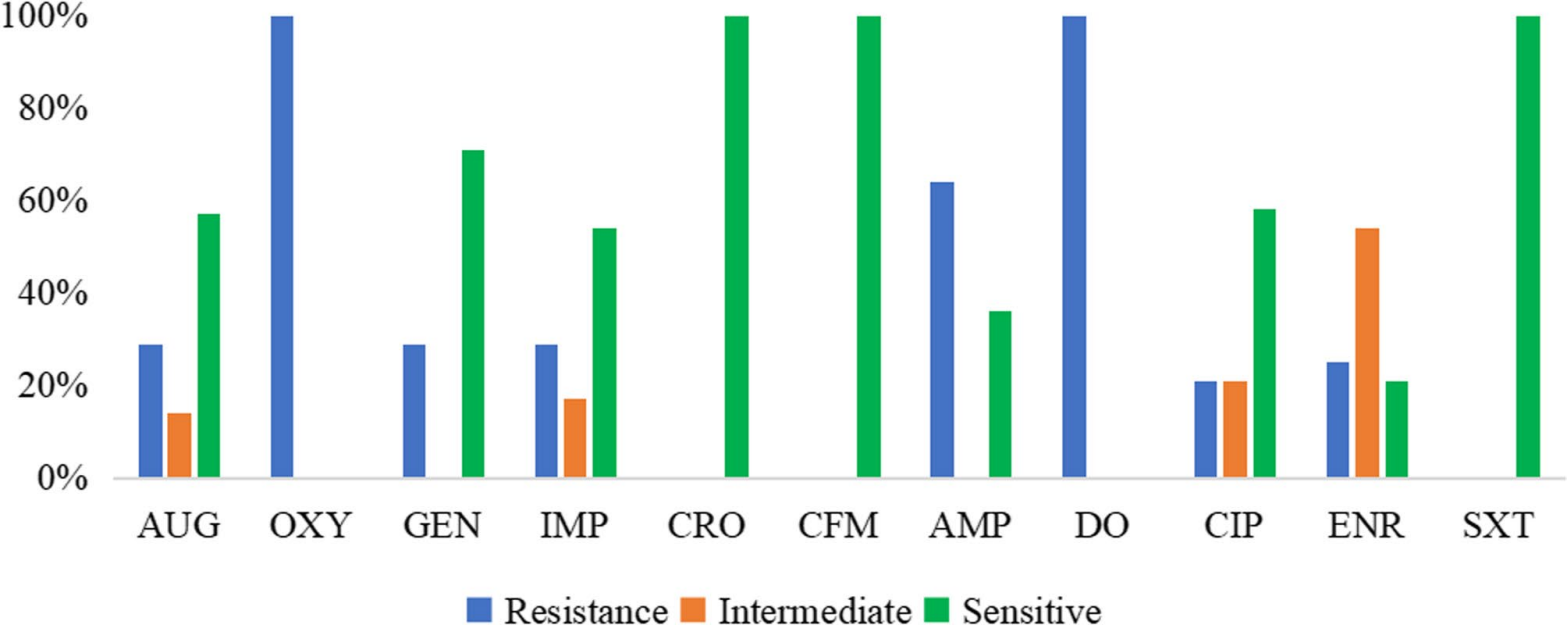
Antimicrobial sensitivity results in 28 *Salmonella*tested strains in this study. Legend: AUG, amoxicillin-clavulanic acid; OXY, oxytetracycline; GEN, gentamicin; IMP, imipenem; CRO, ceftriaxone; CFM, cefixime; AMP, ampicillin; DO, doxycycline; CIP, ciprofloxacin; ENR, enrofloxacin; SXT, trimethoprim-sulfamethoxazole

**Fig. 4:**
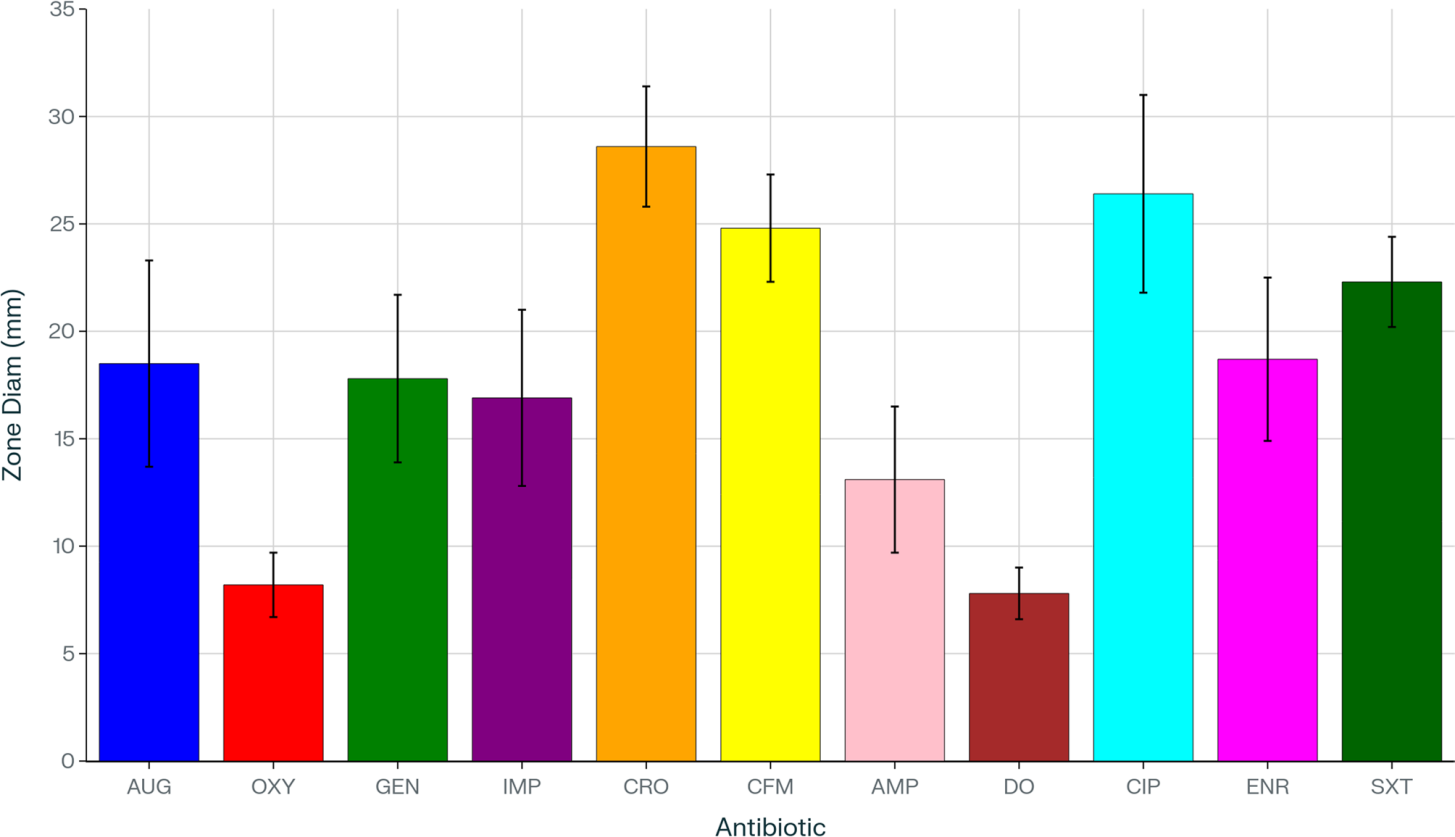
Mean± SD inhibition zone diameters for 11 antibiotics against 28 *Salmonella* strains.Legend: Bar chart displaying the mean inhibition zone diameter (mm) ± standard deviation for each antibiotic (AUG, OXY, GEN, IMP, CRO, CFM, AMP, DO, CIP, ENR, SXT), with multicolored bars on a white background and error bars indicating variability

The antibiotic sensitivity of 28 *Salmonella* isolates was assessed using 11 antimicrobial agents. Complete resistance (100%) was observed against oxytetracycline (8.2 ± 1.5 mm) and doxycycline (7.8 ± 1.2 mm), representing the smallest inhibition zones. High resistance was also noted for ampicillin (64% resistance; 13.1 ± 3.4 mm) and imipenem (29% resistance; 16.9 ± 4.1 mm), while the remaining drugs exhibited low resistance rates (Figs. 3 and 4).

In contrast, three antibiotics achieved complete sensitivity (100%): ceftriaxone (28.6 ± 2.8 mm), cefixime (24.8 ± 2.5 mm), and trimethoprim–sulfamethoxazole (22.3 ± 2.1 mm), demonstrating the largest inhibition zones. High sensitivity was also observed for gentamicin (71% sensitivity; 17.8 ± 3.9 mm) and ciprofloxacin (58% sensitivity; 26.4 ± 4.6 mm). Moderate activity was noted for amoxicillin-clavulanic acid (18.5 ± 4.8 mm) and enrofloxacin (18.7 ± 3.8 mm) (Figs. 3 and 4). The quantitative disc-diffusion data, presented as mean inhibition zone diameters ± SD, clearly demonstrate the correlation between resistance percentages and zone measurements, with resistant antibiotics showing significantly smaller zones compared to sensitive ones (Figs. 3 and 4).

### Detection of antibiotic resistance genes

#### Tetracycline resistance genes

The presence of the *tetA* and *tetC* genes, which confers resistance to tetracycline, was investigated in the *Salmonella* isolates. Agarose gel electrophoresis showed a 494 bp band, confirming the *tetA* gene presence. The molecular weight marker (M) verified the PCR product size (Fig. 2E). The positive *tetC* gene was confirmed by a 418 band in agarose gel electrophoresis shown in Fig. 2B. The *tetA* gene was detected in 25% (7/28) strains and *tetC* gene was identified in only one strain. None any isolates had *tetB*,* tetD*,* tetE*, and *tetG* genes (Table 2).

**Table 2:** Phenotypic resistance patterns and resistance gene profiles of 28 *Salmonella* isolates

Isolate Number	Phenotypic resistance pattern	Number of antibiotics	Antibiotic resistance genes
1	OXY, AMP, DO	3	Nil
2	OXY, AMP, DO	3	*tetA*
3	OXY, AMP, DO	3	*tetA*
4	OXY, IMP, AMP, DO	4	Nil
5	OXY, AMP, DO, ENR	4	Nil
6	OXY, AMP, DO, IMP, CIP, ENR	6	*tetA*,* tetC*,* bla*_TEM−1_
7	OXY, AMP, DO, CIP	4	Nil
8	OXY, CIP, DO	3	Nil
9	AUG, OXY, GEN, DO	4	Nil
10	OXY, GEN, DO	3	*tetA*
11	AUG, OXY, GEN, AMP, DO	5	*bla*_TEM−1_
12	AUG, OXY, GEN, AMP, DO	5	*bla*_TEM−1_
13	OXY, DO	2	Nil
14	OXY, IMP, DO, CIP	4	Nil
15	OXY, DO	2	Nil
16	AUG, OXY, DO, ENR	4	Nil
17	AUG, OXY, AMP, DO, ENR	5	*tetA*,* bla*_TEM−1_
18	OXY, AMP, DO, ENR	4	Nil
19	OXY, AMP, IMP, DO	4	Nil
20	OXY, AMP, IMP, DO	4	*bla*_TEM−1_
21	OXY, IMP, DO	3	Nil
22	DO, IMP, AMP, OXY	4	Nil
23	AUG, OXY, AMP, DO	4	Nil
24	OXY, IMP, DO	3	Nil
25	AUG, OXY, AMP, DO, ENR	5	*tetA*
26	AUG, OXY, AMP, DO	4	Nil
27	OXY, IMP, DO	3	Nil
28	AUG, OXY, AMP, DO, ENR	5	*tetA*

#### Identification of β-lactamase genes

The presence of the *bla*_TEM−1_gene, β-lactamase primarily confers resistance to penicillins, including ampicillin, was searchedin the *Salmonella* isolates. Agarose gel electrophoresis displayed a band of 535 bp, indicating the presence of the *bla*_TEM−1_gene. The molecular weight marker (M) confirmed the size of the PCR product (Fig. 2D). The *bla*_TEM−1_ gene was found in 17.9% (5/28) strains. Other β-lactamase genes *bla*_PSE−1_, *bla*_CMY−2_, and *bla*_OXA_ were studied among the strains and no isolate had any of these genes (Table 2).

#### Investigation of quinolones resistance genes

**Fig. 5:**
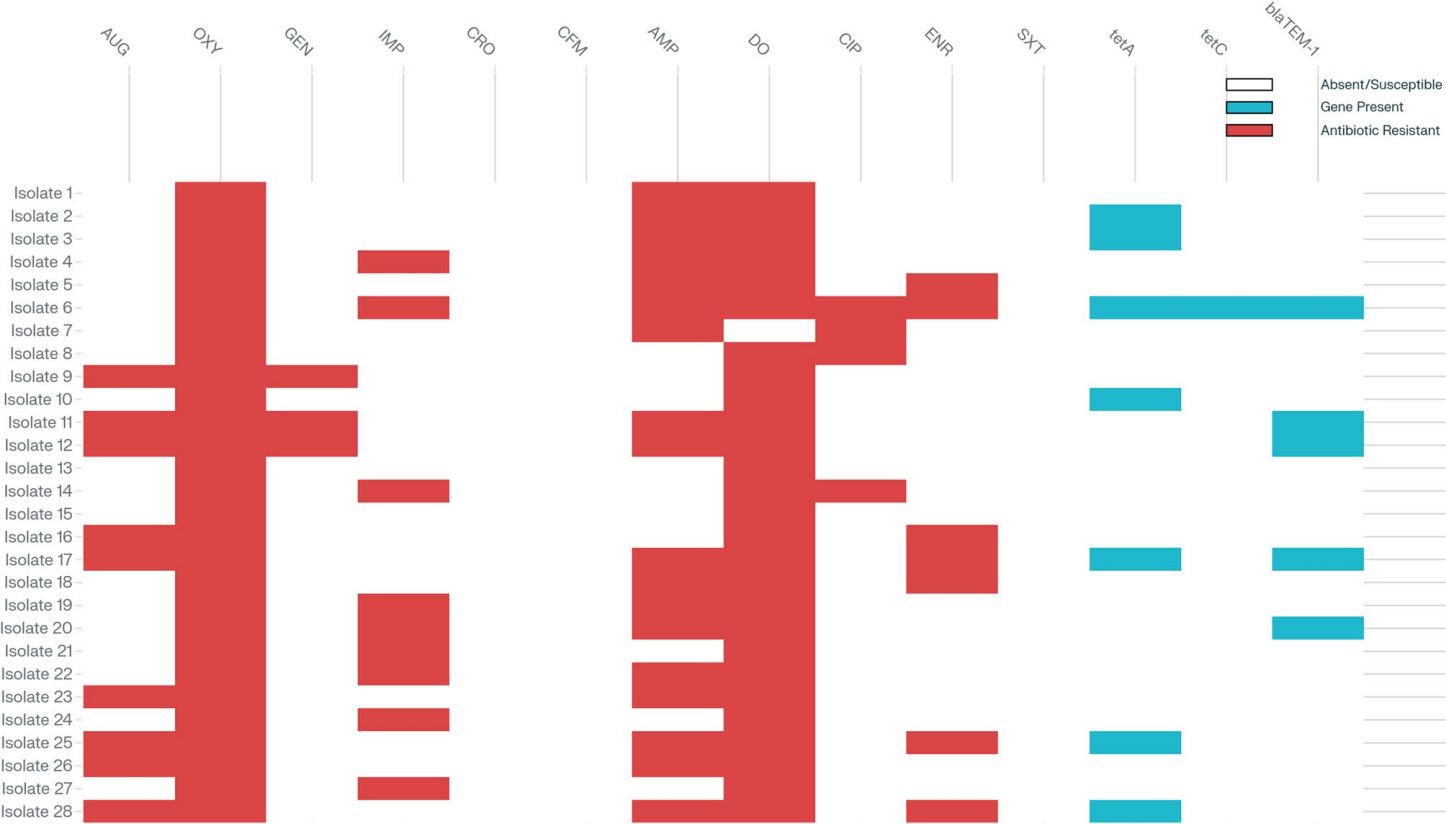
Heatmap of phenotypic resistance and resistance gene presence in 28 *Salmonella* isolates.Legend: Heatmap illustrating phenotypic resistance (red) across 11 antibiotics (AUG, OXY, GEN, IMP, CRO, CFM, AMP, DO, CIP, ENR, SXT) and the presence of resistance genes (*tetA*, *tetC*, *blaTEM-1*; blue). White cells denote susceptibility or gene absence

Screening for plasmid-mediated quinolone resistance determinants (qnrA, qnrB, qnrC, qnrS) yielded no positives across the 28 *Salmonella* isolates. Detailed phenotypic and genotypic profiles are presented in Table 2 and visualized in Fig. 5.

Multidrug resistance, defined as resistance to three or more antimicrobial classes, was observed in 26 of 28 isolates (92.9%). The most frequent phenotypic pattern combined resistance to oxytetracycline (OXY), ampicillin (AMP), and doxycycline (DO). Of these, isolates 2 and 3 specifically carried the *tetA* gene, while isolate 6 showed broad resistance (OXY, AMP, DO, imipenem [IMP], ciprofloxacin [CIP], enrofloxacin [ENR]) and harbored *tetA*, *tetC*, and *blaTEM-1*. β-Lactam resistance was further driven by the *blaTEM-1* gene, detected in 5 isolates (17.9%), including all strains resistant to AMP and amoxicillin–clavulanate (AUG). Several isolates exhibiting resistance to six antibiotics also possessed multiple resistance determinants, underscoring the co-occurrence of tetracycline and β-lactamase genes in highly resistant strains. This comprehensive presentation highlights the absence of quinolone resistance genes despite widespread multidrug resistance mediated by other mechanisms.

## Discussion

*Salmonella* bacteria, particularly *S. enteritidis* and *S. typhimurium*, are identified as the leading cause of food-borne illnesses in the European Union. The emergence of antibiotic resistance in these pathogenic bacteria has become a major concern for public health, leading to an increase in hospitalized patients who fail to respond to treatment. Furthermore, the dissemination of antibiotic resistance genes has resulted in restrictions on the international trade of products. Recent studies have documented antimicrobial resistance in *Salmonella* infections in Pakistan [23, 46]. However, there is a lack of data on antimicrobial resistance in *Salmonella* isolates obtained from animals. Therefore, objective of the current study was to investigate the susceptibility of *Salmonella* isolates to different antibiotics and determine the presence of antibiotic resistance genes. By examining the antibiotic resistance profiles of the isolates, valuable insights can be gained regarding the effectiveness of various antibiotics in treating *Salmonella* infections.

The prevalence of *Salmonella* spp. in fecal samples from cattle was found to be 28%, which aligns with percentages obtained from raw meat products in other studies [3, 14, 47] and is higher than previous studies in Ethiopia, where *Salmonella* spp. was found in 7.3% of fecal samples in dairy cattle [50], and in India, where the prevalence of *Salmonella* was reported at 1.2% (6/508) [43]. In this study, 28 pure isolates of *Salmonella* were tested against 11 different antibiotics to assess their sensitivity and resistance. The results showed that *Salmonella* isolates exhibited full resistance to oxytetracycline and doxycycline, while 65% of the isolates were resistant to ampicillin. A resistance rate of 30% was observed against imipenem. Interestingly, the findings of this study are consistent with another study conducted in Pakistan, which reported 65% resistance to ampicillin, 88% to tetracycline, and 25% to meropenem (carbapenem) among *Salmonella* spp. from raw meat [16], differing from those reported by Su et al., [49], where *Salmonella* was found to be most resistant to ampicillin along with four other tested antibiotics.

In this study, sixteen strains were reported as MDR *Salmonella* (resistant to three or more families of antibiotics). Numerous recent investigations have reported MDR *Salmonella* in raw meat and fecal samples of cattle in Pakistan and other countries [12, 16, 18]. There has been a global increase in the incidence of MDR *Salmonella* in the past few decades. Compared to those infected with susceptible strains, people infected with MDR *Salmonella* strains have been found to have a higher burden of morbidity, longer hospital stays, and a higher mortality rate [12, 26].

Previous study performed by Adesiji et al., [1] reported the presence of *tetA* and *tetC* resistance genes in all tetracycline-resistant *Salmonella* isolates, with a 66.7% resistance rate to tetracycline, the highest in their study. Chuanchuen & Padungtod [10, 37], also reported a higher prevalence of the *tetA* gene compared to the *tetB* gene in *Salmonella* isolates, consistent with our findings where the *tetA* gene was 25% more prevalent than the *tetB* gene. It is worth noting that not all *Salmonella* isolates in our study contained the *tetA* or *tetB* gene. Yasmin et al., [55]reported that resistance to ampicillin, tetracycline, and chloramphenicol antibiotics is common in *Salmonella* spp., aligning with our findings of resistance to ampicillin and tetracyclines.

In the present study, the *Salmonella* strains were screened for β-lactamase genes *bla*_TEM−1_, *bla*_PSE−1_, *bla*_CMY−2_, and *bla*_OXA_. The PCR results indicated that the *bla*_TEM−1_ gene was found in 18% of strains, supported by other studies in Pakistan [3, 12] detecting a high rate of the *bla*_TEM−1_ gene. The presence of β-lactamase determinants in *Salmonella* strains demonstrates that these bacteria possess antibiotic resistance genes to enhance their survival in environments with selective pressure from antimicrobials [25]. This phenomenon is exacerbated by the high consumption of antibiotics in animal farms for prophylaxis, treatment, and growth promotion, ultimately leading to the emergence of multidrug-resistant isolates [38]. In addition to indicating exposure to antimicrobial selection pressure, the presence of β-lactamase genes may also indicate a role for plasmid-mediated transfer, which speeds up the spread of resistance among bacteria. The proliferation of multidrug-resistant *Salmonella* strains is made worse by such transportable genetic factors and the heavy use of antibiotics in cattle [29]. Indeed, a major factor in the current worldwide threat of antibiotic resistance is plasmid-mediated gene spread [48].

Although some antibiotics showed decreased sensitivity during the trial period, they still demonstrated effectiveness and are worth reporting. Bacci et al., [5, 10] reported a very low prevalence rate of the *pse-1* gene, consistent with our study where the *pse-1* gene was not detected. South African studies have indicated the presence of the *pse-1* gene in aquatic systems and livestock production, suggesting an increasing prevalence of β-lactamase in pathogenic bacteria.

From 2000 to 2002, 50% of isolated *Salmonella* strains were found to be resistant to several antibiotics, including amoxicillin, ampicillin, cefoxitin, ceftiofur, ceftriaxone, chloramphenicol, and tetracycline [50]. Resistance to these antibiotics, as well as reduced sensitivity to ceftriaxone, is characteristic of strains producing β-lactamase encoded by the gene *bla*_CMY−2_ [3, 16, 25, 38]. These strains are resistant to cephalothin, cephalosporins, and cephamycins. OH et al., [45] reported higher resistance rates of *Salmonella* spp. to antibiotics such as ampicillin and nalidixic acid. In their study, *S. typhimurium* exhibited the highest resistance rate (85%) to tetracycline among the tested antibiotics. Kayode et al., [24] also reported high levels of resistance (66.7%, 60%, 53.3%, and 50%) to trimethoprim-sulfamethoxazole, tetracycline, and gentamicin, respectively. Similarly, Mwambene et al. [42] found that resistance to antibiotics such as ampicillin, tetracycline, and chloramphenicol is common in *Salmonella* species, which aligns with our study where resistance to these antibiotics was observed.

Resistance to fluoroquinolones, specifically ciprofloxacin (21%) and norfloxacin (25%), was reported in this study, which agrees with an Ethiopian study that found 30% of the isolates resistant to ciprofloxacin [39]. In contrast, Ishaku et al., [21] reported that all (100%) clinical *Salmonella* spp. from northern Nigeria were resistant to fluoroquinolones. However, all clinical *Salmonella* isolated from stool in Montenegro were sensitive to ciprofloxacin and most commonly used antibiotics [36]. Even though our study found 21% of the strains resistant to ciprofloxacin, fluoroquinolones are not commonly used in veterinary medicine in Pakistan, suggesting that the resistance in *Salmonella* spp. is alarming. According to Angulo et al., [4], ciprofloxacin is considered the most important fluoroquinolone for treating salmonellosis in Uganda and other countries. However, in our study, the *QnrS* gene was not found in any of the *Salmonella* isolates. During the late 1990 s, fluoroquinolones were expected to be the treatment of choice for typhoid due to their high effectiveness and minimal side effects.

It is worth noting that fluoroquinolones are not typically used to treat children; however, the occurrence of quinolone-resistant strains in individuals under 15 years of (9.5%) is concerning and may reflect indirect exposure through environmental sources, food chains, or circulation of resistant strains within the community. Quinolone resistance has increased not only in foodborne *Salmonella* but also in Campylobacter infections. As stated by the Minister of Health of the Republic of Indonesia, ampicillin, chloramphenicol, or trimethoprim-sulfamethoxazole are the first-line antibiotics used, and if resistance is detected, second-line antibiotics such as ceftriaxone, cefixime, or quinolones should be considered [19, 32]. In this study, some isolates demonstrated resistance to five or six antibiotics, harboring multiple resistance determinants such as (*tetA*, *tetC*, *bla*_TEM−1_). Overall, these findings highlight the high burden of tetracycline resistance (both phenotypic and genotypic) and the emergence of β-lactamase producing strains, underscoring serious concerns regarding the spread of multidrug-resistant isolates. Additionally, the co-occurrence of β-lactam and tetracycline resistance raises severe implications for public and animal health as it shows the possibility of co-selection and horizontal gene transfer.

## Conclusion

This study provides important insights into the antimicrobial resistance profiles of Salmonella isolates recovered from cattle fecal samples, with a prevalence of 28%. The isolates exhibited complete resistance to tetracycline and doxycycline, along with substantial resistance to ampicillin and imipenem. In contrast, all isolates remained fully susceptible to ceftriaxone, cefixime, and trimethoprim-sulfamethoxazole, suggesting these agents as potentially effective treatment options. Notably, 93% of isolates displayed multidrug resistance (MDR), with some resistant to as many as six antibiotics and carrying multiple resistance genes (*tetA*, *tetC*, *bla*_TEM−1_). The co-occurrence of these genes raises serious concerns about co-selection and horizontal gene transfer, which could accelerate the spread of resistance within bacterial populations. The absence of *qnr* genes, despite fluoroquinolone resistance, further points to alternative mechanisms driving resistance. Overall, the findings highlight the alarming burden of tetracycline resistance and the emergence of β-lactamase-producing MDR Salmonella strains. Continuous AMR surveillance, prudent antimicrobial use in food animals, and strengthened One-Health based interventions are urgently needed to reduce the risk of zoonotic transmission through food, the environment, or direct contact. Additionally, ongoing monitoring of resistance trends across time and regions remains critical to inform treatment guidelines and implement effective control strategies.

## Supplementary Information

Supplementary material 1.

Supplementary material 2.

Supplementary material 3.

## Data Availability

All data generated or analyzed during this study are included in the article.

## Abbreviations

NARC: National Agriculture Research Center Islamabad
MDR: multidrug resistant
H_2_S: hydrogen sulfide
MIC: minimum inhibitory concentration
CLSI: Clinical and Laboratory Standards Institute
PCR: Polymerase Chain Reaction
dNTPs: deoxynucleotide triphosphates
