# Supplementary material for: Antibiotic susceptibility pattern and resistance genes in Salmonella strains isolated from cattle
Source: BMC Vet Res. 2025 Nov 14;21:665. doi: 10.1186/s12917-025-05081-4 (PMC12619517; doi:10.1186/s12917-025-05081-4)
Supplement: Supplementary file 1 — Supplementary material 1. [file 12917_2025_5081_MOESM1_ESM.docx]

| Sr. No. | Antibiotics | Class | Symbol | Concentration |
| --- | --- | --- | --- | --- |
| 1 | Co-Amoxiclav | Penicillin | AUG | 30µg |
| 2 | Oxytetracycline | Tetracycline | OTC | 30µg |
| 3 | Gentamicin | Aminoglycoside | GEN | 10µg |
| 4 | Imipenem | Carbapenem | IMP | 10µg |
| 5 | Ceftrioxone | Cephalosporin | CRO | 30µg |
| 6 | Cefixime | Cephalosporin | CFM | 5µg |
| 7 | Ampicillin | Penicillin | AMP | 10µg |
| 8 | Doxycycline | Tetracycline | DO | 30µg |
| 9 | Ciprofloxacin | Quinolone | CIP | 5µg |

**Supplementary file 1:** List of antibiotics discs used with their symbols and concentrations
