# Supplementary material for: Antibiotic susceptibility pattern and resistance genes in Salmonella strains isolated from cattle
Source: BMC Vet Res. 2025 Nov 14;21:665. doi: 10.1186/s12917-025-05081-4 (PMC12619517; doi:10.1186/s12917-025-05081-4)
Supplement: Supplementary file 2 — Supplementary material 2. [file 12917_2025_5081_MOESM2_ESM.docx]

**Supplementary file 2:** Reference values of antimicrobial sensitivity results

| **Antimicrobial Agents** | **Resistance (mm)** | **Intermediate (mm)** | **Sensitivity (mm)** |
| --- | --- | --- | --- |
| AUG | ≤13 | 14-17 | ≥18 |
| OTC | ≤11 | 12-14 | ≥15 |
| GEN | ≤12 | 13-15 | ≥16 |
| IMP | ≤16 | 17-20 | ≥21 |
| CRO | ≤23 | 24-27 | ≥28 |
| CFM | ≤15 | 16-18 | ≥19 |
| AMP | ≤13 | 14-16 | ≥17 |
| DO | ≤12 | 13-15 | ≥16 |
| CIP | ≤15 | 16-20 | ≥21 |
| ENR | ≤16 | 17-22 | ≥23 |
| SXT | ≤12 | 13-16 | ≥17 |
