# Supplementary material for: Antibiotic susceptibility pattern and resistance genes in Salmonella strains isolated from cattle
Source: BMC Vet Res. 2025 Nov 14;21:665. doi: 10.1186/s12917-025-05081-4 (PMC12619517; doi:10.1186/s12917-025-05081-4)
Supplement: Supplementary file 3 — Supplementary material 3. [file 12917_2025_5081_MOESM3_ESM.docx]

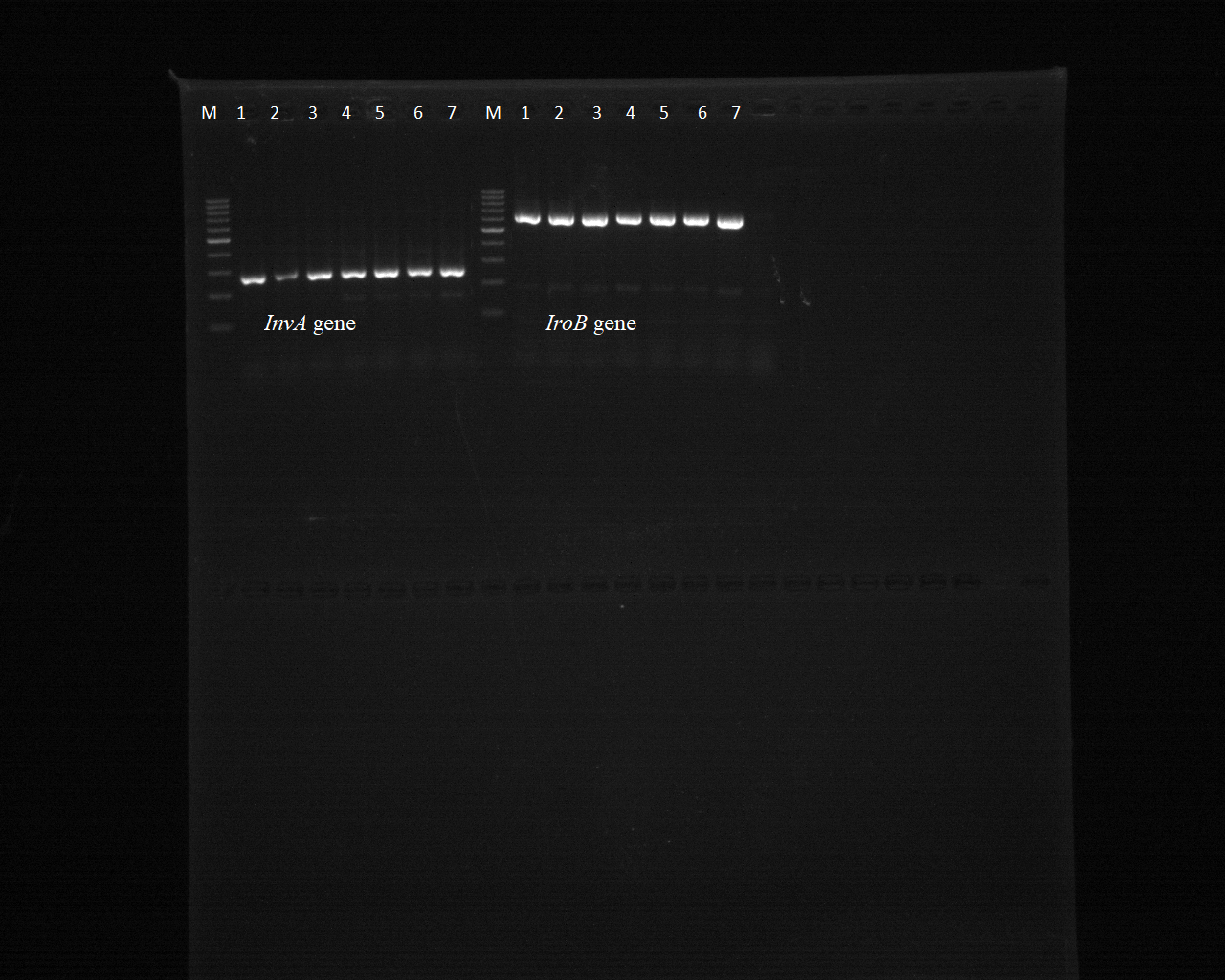


*InvA* gene (284bp), *IroB* gene (606bp)


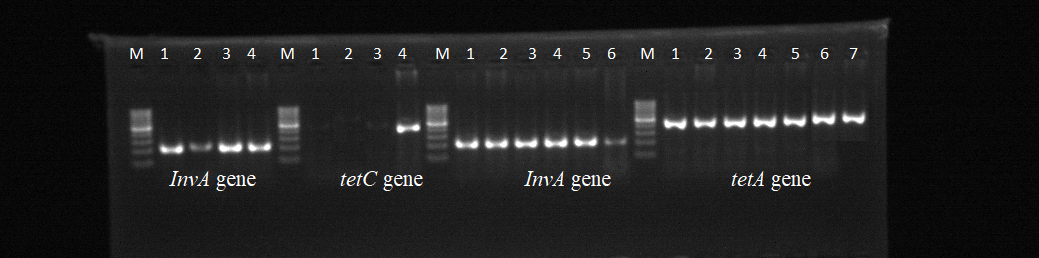


*InvA* gene (284bp), *tetC* gene (418bp), *tetA* gene (494bp)


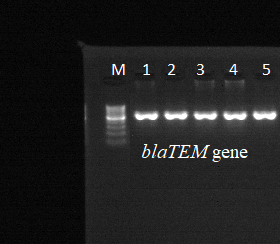


blaTEM gene (535 bp)


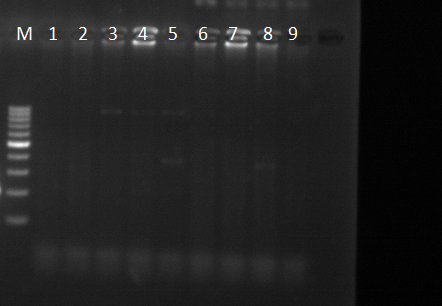


no gene detected for *blaPSE-1, blaCMY-2, blaOXA, qnrA, qnrB, qnrC, qnrS, tetB, tetD, tetE,* and *tetG.*
